# Semaphorin 7A promotes endothelial permeability and inflammation via plexin C1 and integrin β1 in Kawasaki disease

**DOI:** 10.1186/s12887-024-04766-3

**Published:** 2024-04-27

**Authors:** Junhua Huang, Chuanmei Zhao, Shuwan Zhang

**Affiliations:** 1 School of Medical Technology, Xi’an Medical University, Xi’an, 710021 Shaanxi Province China; 2 Department of Clinical Laboratory, Xi’an Children’s Hospital, Xi’an, 710003 Shaanxi Province China

**Keywords:** Kawasaki disease, Semaphorin 7A, Endothelial cell, Inflammation

## Abstract

**Background:**

Kawasaki disease (KD) is a pediatric systemic vasculitis characterized by endothelial cell dysfunction. Semaphorin 7A (Sema7A) has been reported to regulate endothelial phenotypes associated with cardiovascular diseases, while its role in KD remains unknown. This study aims to investigate the effect of Sema7A on endothelial permeability and inflammatory response in KD conditions.

**Methods:**

Blood samples were collected from 68 KD patients and 25 healthy children (HC). The levels of Sema7A and A Disintegrin and Metalloprotease 17 (ADAM17) in serum were measured by enzyme-linked immunosorbent assay (ELISA), and Sema7A expression in blood cells was analyzed by flow cytometry. Ex vivo monocytes were used for Sema7A shedding assays. In vitro human coronary artery endothelial cells (HCAECs) were cultured in KD sera and stimulated with Sema7A, and TNF-α, IL-1β, IL-6, and IL-18 of HCAECs were measured by ELISA and qRT-PCR. HCAECs monolayer permeability was measured by FITC-dextran.

**Results:**

The serum level of Sema7A was significantly higher in KD patients than in HC and correlated with disease severity. Monocytes were identified as one of the source of elevated serum Sema7A, which implicates a process of ADAM17-dependent shedding. Sera from KD patients induced upregulation of plexin C1 and integrin β1 in HCAECs compared to sera from HC. Sema7A mediated the proinflammatory cytokine production of HCAECs in an integrin β1-dependent manner, while both plexin C1 and integrin β1 contributed to Sema7A-induced HCAEC hyperpermeability.

**Conclusions:**

Sema7A is involved in the progression of KD vasculitis by promoting endothelial permeability and inflammation through a plexin C1 and integrin β1-dependent pathway. Sema7A may serve as a potential biomarker and therapeutic target in the prognosis and treatment of KD.

## Background

Kawasaki disease (KD) is an acute systemic vasculitis of unknown etiology and is one of the common acquired heart diseases in children under 5 years of age [1]. The hallmark of KD is vascular damage, with coronary artery lesions (CAL) being the main complication [2]. Approximately 30% of KD children may develop CAL if intravenous immunoglobulin (IVIG) is not administered promptly, leading to a worsened prognosis and higher medical costs [3]. While the exact mechanisms underlying KD vasculitis remain unclear, accumulating evidence suggests that increased permeability and inflammatory responses of endothelial cells are two major features of KD vasculitis, resulting in pathological vascular leakage and immune-inflammatory cell infiltration into the vessel lesions. Numerous molecules are implicated in the processes associated with endothelial hyperpermeability and pro-inflammatory cytokine production in KD. However, few studies have reported on a multifunctional molecule that is involved in various endothelial phenotypic changes, including increased endothelial permeability and enhanced cytokine secretion.

Semaphorin encompasses various transmembrane and secreted proteins and is classified into 8 subclasses based on sequence similarity and distinctive structural features [4]. While it serves as an important axon guidance molecule during neural development, growing evidence suggests that semaphorin also plays important roles in autoimmune diseases, inflammatory disorders, and cardiovascular diseases [5]. Semaphorin 7A (Sema7A) is the only class 7 semaphorin protein anchored to the cell membrane by a glycosylphosphatidylinositol (GPI) moiety and has recently gained attention as an important pro-inflammatory Sema protein [6]. Importantly, in certain disease conditions, GPI-anchored Sema7A can be cleaved by metalloproteinases into soluble Sema7A (sSema7A). Both membrane-bound Sema7A (mSema7A) and sSema7A can exert biological functions through binding to two major receptors, plexin C1 and integrin β1. Sema7A has been reported to mediate endothelial cell destabilization [7], activation of immune cells, and cytoskeletal remodeling [8], all of which are closely associated with vasculitis. However, whether Sema7A is involved in the pathogenesis of KD remains unclear. Here, we propose that Sema7A could participate in the regulation of endothelial function in KD.

## Methods

### Human subjects and ethic statement

Blood samples were collected from 68 patients with acute KD before IVIG treatment, with 22 of them experiencing CAL, and 46 without CAL. Additionally, blood samples were collected from 39 KD patients at the subacute and convalescent stages, respectively. A total of 25 healthy children (HC) were included as control subjects. The diagnosis of KD was based on the criteria outlined in the “Diagnosis, Treatment, and Long-Term Management of Kawasaki Disease” published by the American Heart Association in 2017 [9]. To determine the presence of CAL, we detected the following 7 sites of coronary artery by echocardiography: left main coronary artery (between its opening and the bifurcation of the circumflex branch), left anterior descending-proximal segment (3 ∼ 5 mm after its opening), left circumflex branch (3 ∼ 5 mm after its opening), right coronary artery (RCA)-proximal segment (3 ∼ 5 mm after its opening), RCA-middle segment (right atrioventricular groove), RCA-distal segment (right posterior atrioventricular groove) and posterior descending coronary artery (posterior interventricular groove). When Z-score of one or more of the detected sites in these coronary arteries was ≥ 2, the KD patient was defined as CAL (If Z-score of more than one sites presented ≥ 2, the largest one was recorded). Serum was separated from the blood samples by centrifugation at 3000×*g* for 10 min and stored at -80 °C until further use. Written informed consent was obtained from the parents of all subjects. This study was approved by the Ethics Committee of Xi’an Children’s Hospital and adhered to the principles outlined in the 2013 Declaration of Helsinki.

### Blood assessment

The levels of Sema7A, A Disintegrin and Metalloprotease 17 (ADAM17), and matrix metallopeptidase 9 (MMP9) in serum were measured using enzyme-linked immunosorbent assay (ELISA) kits (Cloud-Clone, USA) following the manufacturer’s instructions. Monocyte counts were analyzed using a blood routine analyzer (Sysmex XS500, Japan). Albumin and C-reactive protein (CRP) levels were assessed using an automatic biochemical analyzer (BeckmanCoulter, USA).

### Flow cytometry (FCM)

Blood samples were analyzed using the NovoCyte D1040 flow cytometer (ACEA, USA) with NovoExpress software (ACEA, USA). The following antibodies were used: PE-conjugated anti-human Sema7A, PECY5-conjugated anti-human CD3, PECY5-conjugated anti-human CD14, and PECY5-conjugated anti-human CD15 (Biolegend, USA).

### Collection and treatment of monocytes

Peripheral blood mononuclear cells (PBMCs) from HC were isolated using density gradient centrifugation with Ficoll-Paque (Sigma-Aldrich, USA). Magnetic beads conjugated with anti-CD14 antibodies (Miltenyi, USA) were then utilized to label and separate monocytes from PBMCs, following the manufacturer’s instructions. The isolated monocytes were cultured in Roswell Park Memorial Institute (RPMI) 1640 medium supplemented with 10% fetal bovine serum (FBS).

For the monocyte Sema7A shedding assay, isolated monocytes (1 × 10^6^ cells per well in a 24-well plate) were pretreated with 10 µg/ml of MMP9 or ADAM17 (R&D Systems, USA) for 30 min, respectively. For the spontaneous Sema7A shedding inhibition assay, TAPI-1 (an ADAM17 inhibitor, Selleck Chemicals) was added to the RPMI 1640 medium for 30 min. Subsequently, sSema7A in the supernatant was assessed using ELISA.

### Culture and treatment of HCAECs

HCAECs obtained from Sciencell (CA, USA) were cultured in RPMI 1640 medium supplemented with 10% FBS or 20% human sera, as previously described [10]. Briefly, HCAECs were cultured in RPMI 1640 medium containing 20% KD serum (KDS) or healthy children serum (HCS) for 6 h to establish a KD cell model. Subsequently, mRNA was extracted from the HCAECs for reverse transcription and quantitative real-time polymerase chain reaction (qRT-PCR) analysis of plexin C1 and integrin β1.

For stimulation with recombinant human Sema7A (rhSema7A) obtained from R&D Systems (USA), after the 6-hour treatment with human sera, HCAECs were washed with phosphate buffer solution (PBS) and the medium was replaced with RPMI 1640 medium containing 10% FBS. Subsequently, 10 µg/ml of rhSema7A was added to the medium, and the cells were incubated for 12 h. The culture supernatants were collected for ELISA analysis of TNF-α, IL-1β, IL-6, and IL-18 using the EliKineTM kits from Abbkine (China). Additionally, mRNA was extracted from the HCAECs for reverse transcription and subsequent qRT-PCR analysis of TNF-α, IL-1β, IL-6, and IL-18.

For the receptor blockade assay, anti-integrin β1 antibody and anti-plexin C1 antibody from Abcam (UK) were added to the medium for 1 h. Afterward, the cells were washed with PBS and stimulated with rhSema7A.

### qRT-PCR

Total RNA was extracted from HCAECs using the RNeasy mini kit (Qiagen, Germany). cDNA was synthesized using the PrimeScript RT Mix (Takara, Japan). The mRNA expression levels of plexin C1, integrin β1, TNF-α, IL-1β, IL-6, and IL-18 were analyzed using SYBR Green Realtime PCR Mix (Takara, Japan) on an ABI 7500 analyzer (ABI, USA).

### Endothelial permeability assays

To assess endothelial permeability, monolayer HCAECs were seeded on transwells, as described previously [11]. Once the monolayer was formed, 200 µl of 300 µg/ml FITC-dextran (molecular weight = 10,000, Sigma) was added to the upper chamber and incubated for 30 min. The fluorescence intensity in the lower chamber, which represents the amount of FITC-dextran that passed through the endothelial monolayer, was measured using a fluorescence microplate reader from PerkinElmer (USA).

### Statistical analyses

The data were presented as the mean ± standard deviation (M ± SD) for continuous variables or as numbers for categorical variables. Student’s t-test was used to compare continuous variables between two groups, while Fisher’s exact test was used for categorical variables. Pearson correlation analysis was conducted to explore the association between sSema7A and other parameters. Statistical analyses were performed using GraphPad Prism 7.0 software (CA, USA), and a *p*-value of less than 0.05 was considered statistically significant.

## Results

### Serum sSema7A is increased in KD patients and correlates with disease severity

**Fig. 1:**
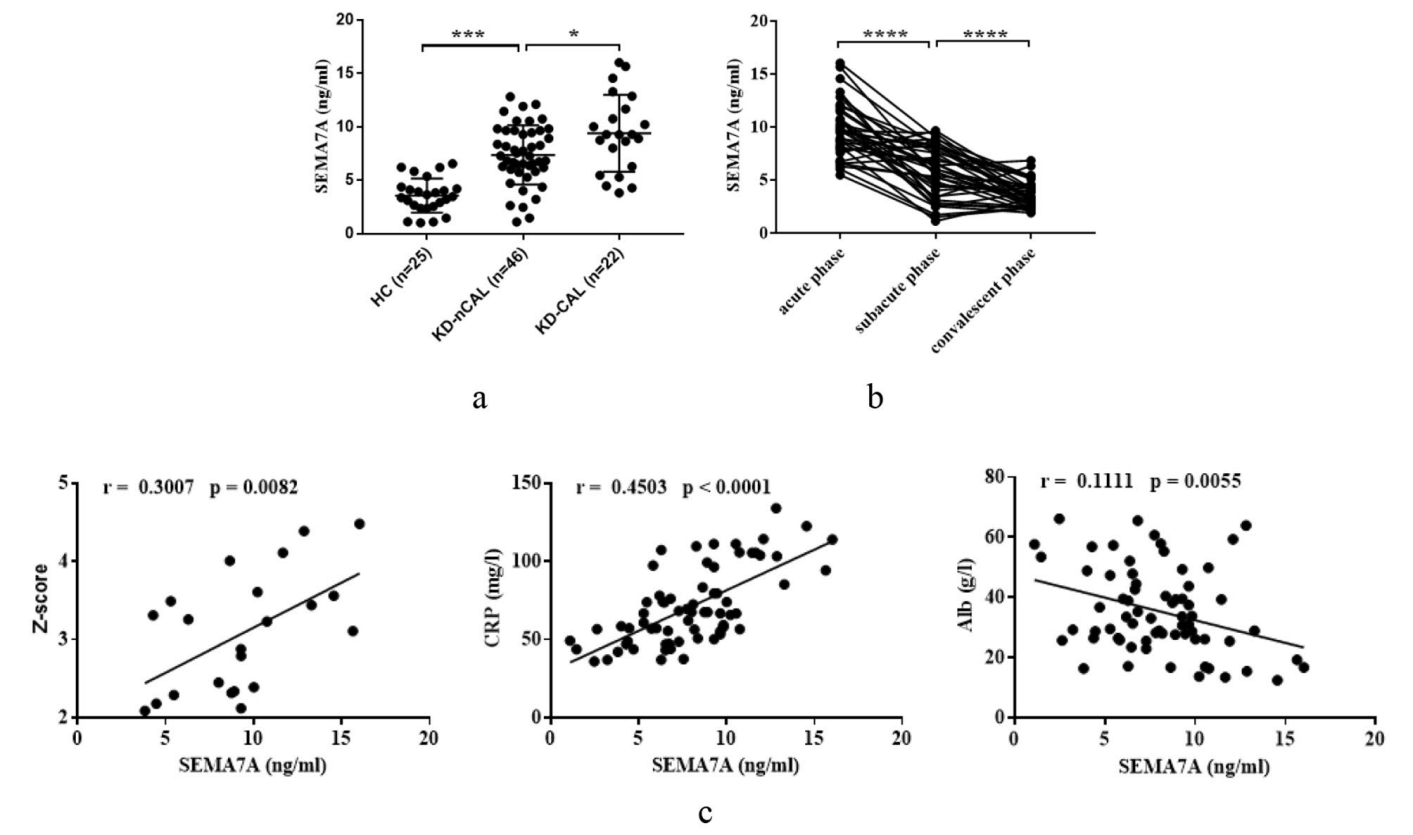
sSema7A is increased in serum of KD patients and correlated with disease severity. **a** Levels of serum sSema7A in HC, KD-nCAL and KD-CAL. **b** Changes of serum sSema7A at the same KD patient at the acute, subacute and convalescent phase (*n* = 39). **c** Relationship between sSema7A and Z-score (*n* = 22), CRP and albumin (*n* = 68). *P < 0.05; ***P < 0.001; ****P < 0.0001. Sema7A: Semaphorin 7A; HC: health control; KD: Kawasaki disease; CAL: coronary artery lesions; nCAL: no CAL; CRP: C reaction protein; Alb: albumin

To investigate the potential involvement of Sema7A in the pathological process of KD, the concentration of sSema7A was assessed in the serum of KD patients. The results, shown in Table 1; Fig. 1a, revealed that the level of sSema7A was significantly higher in the serum of KD patients compared to HC, particularly in patients with CAL. Interestingly, after IVIG treatment, the sSema7A level decreased notably in the subacute phase and further declined to a normal level in the convalescent phase (Fig. 1b). To further determine the clinical significance of sSema7A, the relationship between sSema7A concentration and the levels of Z-score (used to assess coronary artery dilation) and CRP was investigated. The results showed a positive correlation between sSema7A and Z-score as well as CRP levels. Additionally, as KD is characterized by hypoalbuminemia due to albumin leakage outside the blood vessels [12] and reduced hepatic synthesis in the face of the acute phase response [13], we also investigated the relationship between sSema7A and albumin. The results showed a negative correlation between the two variables (Fig. 1c). Taken together, our findings suggest a potential pathological role of sSema7A in KD vasculitis and indicate a close association between Sema7A and the severity of KD.

**Table 1:** Comparison of baseline characteristics between KD with or without CAL and HC

Groups	HC	KD-nCAL	KD-CAL
Number	25	46	22
Male	15	29	14
Female	10	17	8
Age (months)	35.34 ± 21.30	33.15 ± 17.76	30.45 ± 15.58
Z-score (Median)	-	-	3.17
Monocyte counts (10^9^/l)	0.60 ± 0.29	0.82 ± 0.44*	1.11 ± 0.55^#^
CRP (mg/l)	12.25 ± 3.90	65.89 ± 23.79*	82.78 ± 22.82^#^
Alb (g/l)	47.48 ± 11.31	38.80 ± 13.16*	28.16 ± 14.36^#^
ADAM17 (pg/ml)	242.70 ± 67.87	327.80 ± 86.36*	394.40 ± 127.30^#^
MMP9 (ng/ml)	21.50 ± 4.75	40.57 ± 15.40*	45.21 ± 19.61*
Sema7A (ng/ml)	3.57 ± 1.60	7.38 ± 2.77*	9.41 ± 3.60^#^

Data are expressed as M ± SD for quantitative variables or number for categorical variables. *P < 0.05 versus HC. ^#^*P* < 0.05 versus KD-nCAL. KD: Kawasaki disease; CAL: coronary artery lesions; HC: health controls; nCAL: no CAL; CRP: C reaction protein; Alb: albumin; ADAM17: A disintegrin and metalloproteinase domain 17; MMP9: matrix metalloproteinase 9; Sema7A: Semaphorin 7A

### ADAM17-mediated Sema7A shedding from monocytes may be responsible for the increase of serum sSema7A in KD

**Fig. 2:**
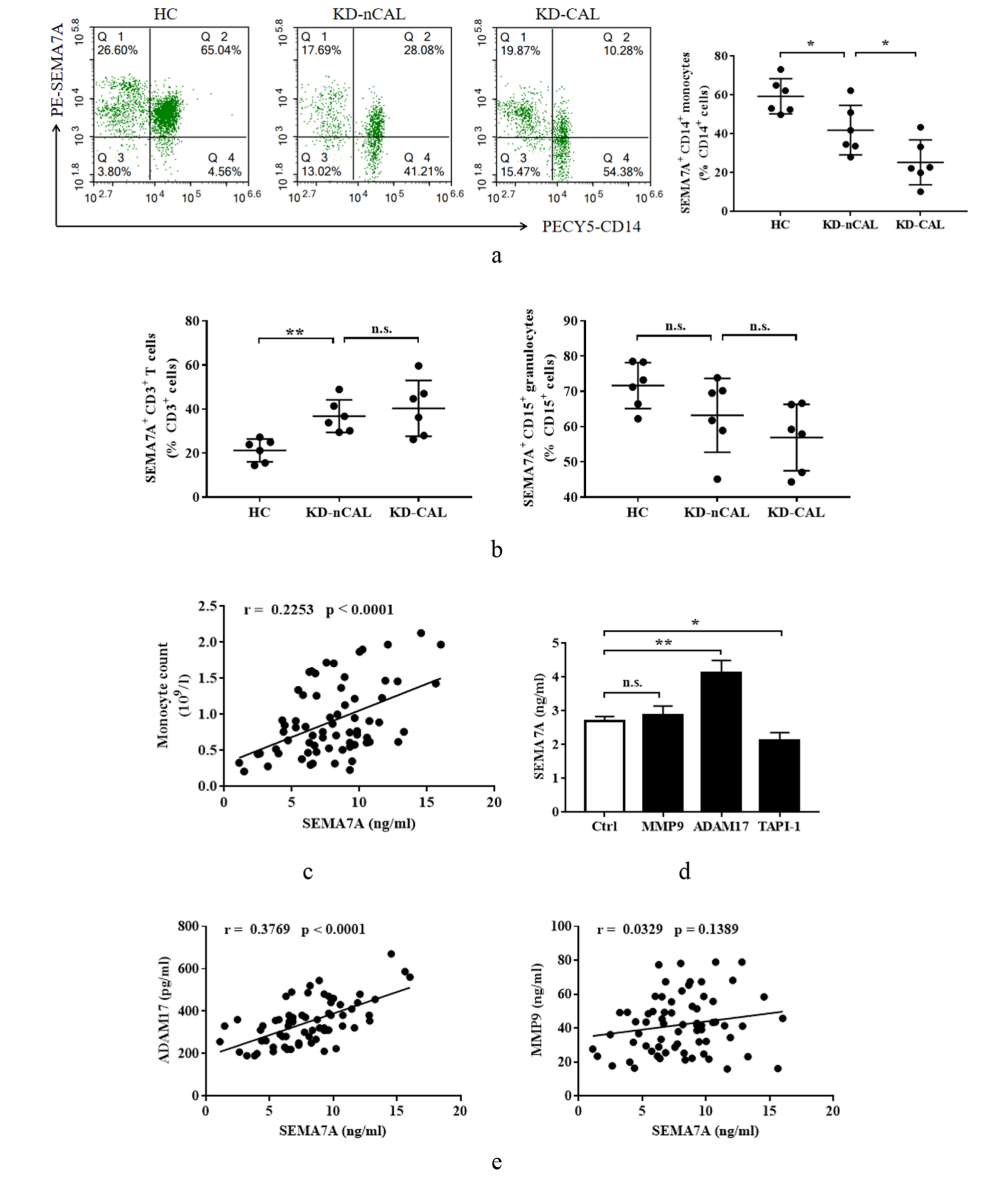
Monocyte mSema7A shedding mediated by ADAM17 may be one of the reasons of increased serum sSema7A in KD. **a** Percentage of Sema7A^+^ monocytes in CD14^+^ cells. The result shown is representative of FCM findings from KD-nCAL (*n* = 6), KD-CAL (*n* = 6) and HC (*n* = 6). **b** Percentage of Sema7A^+^ cells in CD3^+^ T cells and CD15^+^ granulocytes from KD-nCAL (*n* = 6), KD-CAL (*n* = 6) and HC (*n* = 6). **c** The relationship between serum sSema7A concentration and monocyte counts in KD (*n* = 68). **d** Concentration of sSema7A in the culture supernatant of healthy donor monocytes treated with MMP9, ADAM17 and TAPI-1 (a specific ADAM17 inhibitor). Data are expressed as mean ± standard error of mean (M ± SEM) from 3 separate experiments. **e** The relationship of sSema7A with ADAM17 and MMP9 in the sera of KD patients (*n* = 68). *P < 0.05; **P < 0.01; n.s.: not significant. Sema7A: Semaphorin 7A; HC: health control; KD: Kawasaki disease; CAL: coronary artery lesions; nCAL: no CAL; MMP9: matrix metalloproteinase 9; ADAM17: A disintegrin and metalloproteinase domain 17

To investigate the underlying mechanism behind the elevation of serum sSema7A in KD, we examined the cell surface expression of Sema7A on circulating leukocytes using FCM. The results, presented in Fig. 2a and b, demonstrated that the expression of mSema7A on CD14^+^ monocytes, but not on CD15^+^ granulocytes or CD3^+^ T cells, was significantly lower in KD patients compared to HC. Additionally, we found a positive correlation between the concentration of sSema7A in the serum and the circulating monocyte counts (Fig. 2c). Together, these observation suggested that monocytes partly contribute to the increased levels of sSema7A in the serum of KD patients.

Metalloproteases, including MMP9 and ADAM17, have been implicated in the cleavage of mSema7A in certain disease conditions [14]. Both MMP9 and ADAM17 have also been reported to be involved in the development of KD [15]. Therefore, we sought to determine which metalloproteinase was responsible for the shedding of mSema7A from monocytes. As shown in Fig. 2d, stimulation with ADAM17 significantly increased the sSema7A level in the culture supernatant of healthy human monocytes, while MMP9 did not have the ability to promote monocyte mSema7A shedding. Furthermore, we observed that TAPI-1, a specific inhibitor of ADAM17, suppressed the spontaneous shedding of mSema7A on healthy monocytes. Interestingly, we also found a positive correlation between sSema7A and ADAM17, but not MMP9, in the blood of KD patients (Fig. 2e). Taken together, these findings suggest that ADAM17-mediated Sema7A shedding from monocytes may be responsible for the increase in serum sSema7A levels observed in KD.

### Sera from KD patients upregulate the expression of Sema7A receptors in HCAECs

**Fig. 3:**
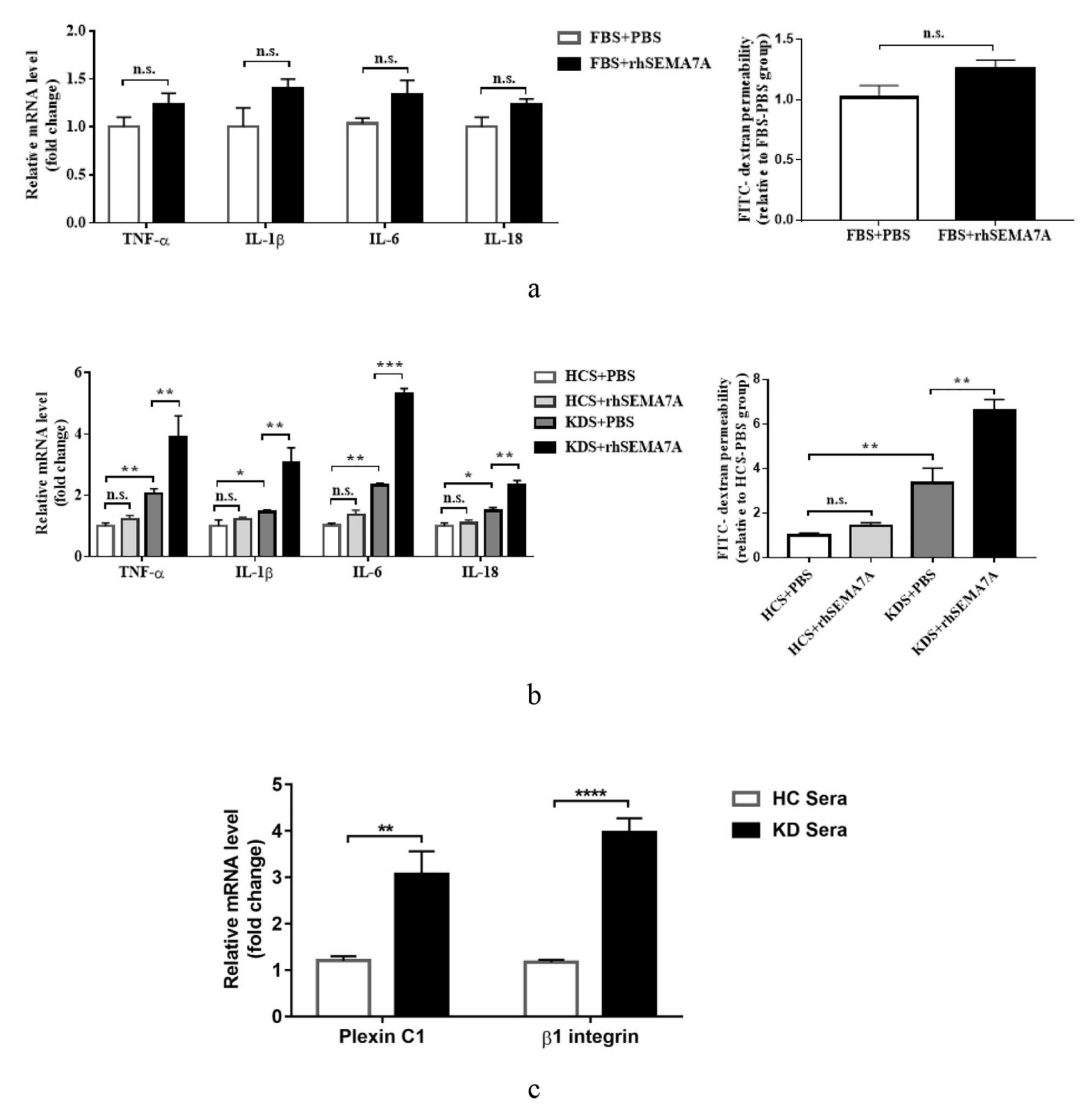
KD sera upregulate the expression of Sema7A receptors in HCAECs. **a** The proinflamamtory cytokine mRNA expression and permeability of HCAECs cultured in 10% FBS-containing RPMI 1640 and subsequently stimulated with 10 µg/ml rhSema7A or not. **b** The proinflamamtory cytokine mRNA expression and permeability of HCAECs cultured in 20% KD sera-containing or 20% HC sera-containing RPMI 1640 and subsequently stimulated with 10 µg/ml rhSema7A or not. **c** The mRNA expression levels of plexin C1 and integrin β1 in HCAECs treated with 20% KD sera or HC sera. Data are expressed as M ± SEM from 3 separate experiments. *P < 0.05; **P < 0.01; ***P < 0.001; ****P < 0.0001; n.s.:not significant. Sema7A: Semaphorin 7A; FBS: fetal bovine serum; PBS: phosphate buffered saline; KDS: sera from KD patients (RPMI 1640 containing 20% KD sera (pooled from 10 KD patients) ); HCS: sera from healthy controls (RPMI 1640 containing 20% healthy control sera (pooled from 10 healthy controls) ); KD: Kawasaki disease

Sema7A has been reported to act as a damaging factor for endothelial cells [16]. Here, we aimed to investigate whether Sema7A is also involved in the damage or activation of HCAECs. We stimulated HCAECs cultured in RPMI 1640 medium containing 10% FBS with rhSema7A and evaluated the expression of inflammatory cytokines and the permeability of HCAEC monolayers. Contrary to our expectations, as shown in Fig. 3a, there were no significant differences in the effects between rhSema7A and PBS on the phenotypic changes of HCAECs, although there was a slight increase in inflammatory cytokine expression and HCAEC monolayer permeability in the rhSema7A stimulation group. A study by Hu et al. [7] suggested that Sema7A is expressed at almost undetectable levels in normal mouse endothelial cells, and endothelial Sema7A expression can be upregulated in certain disease conditions. Additionally, studies from other research teams [17] and our previous article [10] have demonstrated that sera from KD patients can cause alterations in the expression of certain molecules in endothelial cells, and specifically, semaphorins and their receptors can also be changed in the context of inflammation [18]. Based on this evidence, we speculated that Sema7A may exert its proinflammatory roles in a KD inflammatory environment where endothelial cell phenotypes have been altered. To test this hypothesis, we pretreated HCAECs with 20% sera from KD patients followed by rhSema7A stimulation. Surprisingly, we found that compared to pretreatment with 20% sera from HC, the expression of inflammatory cytokines and the monolayer permeability were significantly increased in HCAECs pretreated with 20% sera from KD patients, and rhSema7A further exacerbated these effects specifically in the context of KD but not in normal conditions (Fig. 3b). Furthermore, to understand the underlying mechanism behind this phenomenon, we investigated the impact of KD sera on the expression of Sema7A receptors. Strikingly, we found that KD sera significantly upregulated the expression of plexin C1 and integrin β1 (Fig. 3c).

### Sema7A mediates cytokine production of HCAECs by binding to integrin β1

**Fig. 4:**
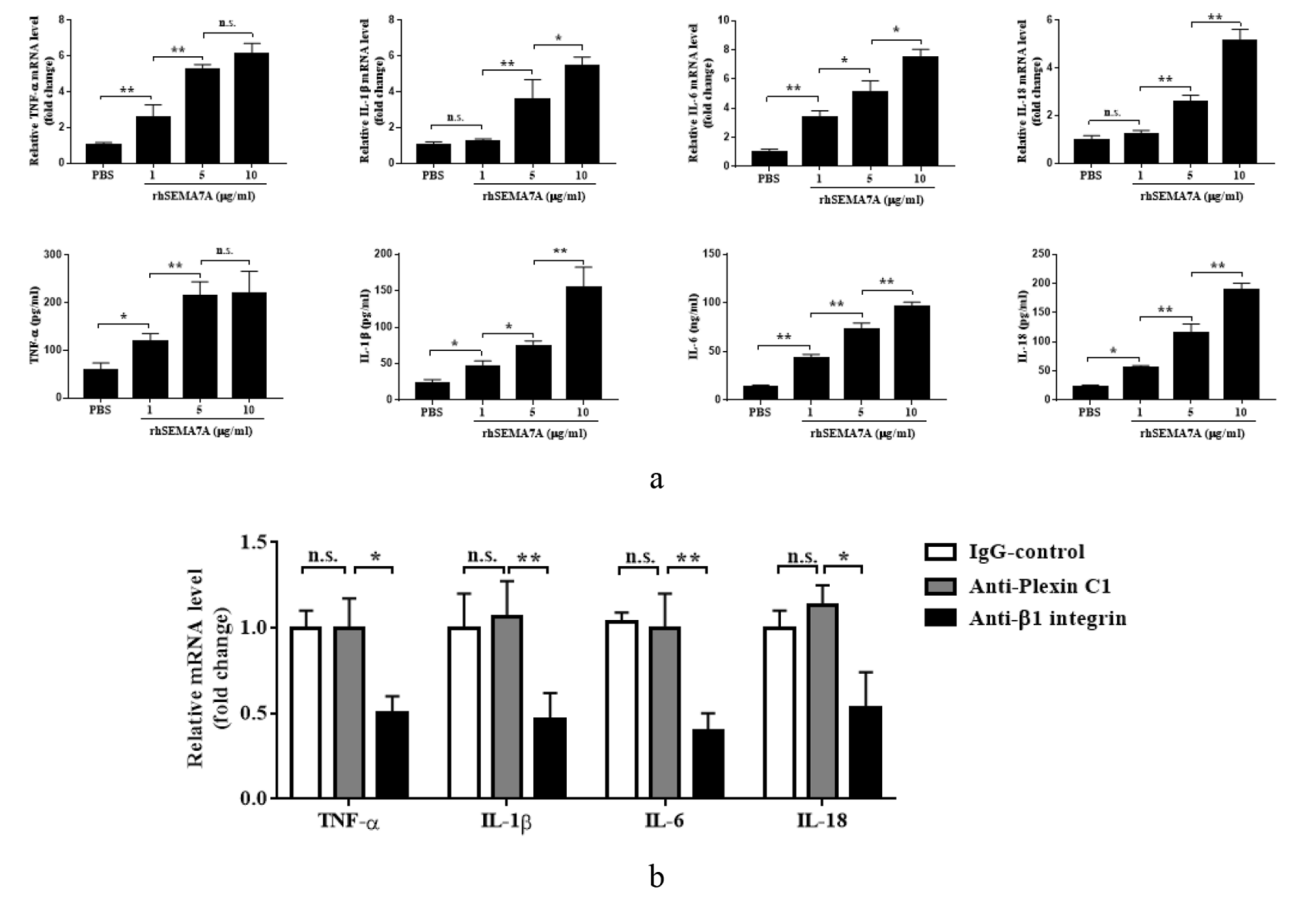
Sema7A mediates cytokine production of HCAECs by binding to integrin β1. **a** The mRNA expression levels and culture supernatant concentrations of TNF-α, IL-1β, IL-6 and IL-18 of HCAECs stimulated with different dosage of rhSema7A. **b** The mRNA expression levels of TNF-α, IL-1β, IL-6 and IL-18 of HCAECs pretreated with anti-plexin C1 antibody and anti-integrin β1 antibody, respectively, followed by 10 µg/ml rhSema7A stimulation. Data are expressed as M ± SEM from 3 separate experiments. *P < 0.05; **P < 0.01; n.s.:not significant. Sema7A: Semaphorin 7A; TNF: tumor necrosis factor; IL: interleukin

Plexin C1 and integrin β1, as specific receptors of Sema7A, have been reported to be involved in the disruption of endothelial cell homeostasis, and both receptors are upregulated in HCAECs treated with KD sera (as shown in Fig. 3c). To determine which receptor contributes to cytokine production in HCAECs under KD conditions, we first cultured HCAECs in RPMI medium containing 20% KD sera and subsequently stimulated them with different doses of rhSema7A. Our results demonstrated that rhSema7A dose-dependently increased the overexpression of TNF-α, IL-1β, IL-6, and IL-18 in HCAECs (Fig. 4a). Next, we pretreated HCAECs cultured in a medium containing 20% KD sera with anti-plexin C1 antibody and anti-integrin β1 antibody, respectively, and then stimulated the HCAECs with rhSema7A. As shown in Fig. 4b, the mRNA overexpression of TNF-α, IL-1β, IL-6, and IL-18 induced by rhSema7A was significantly reduced in the presence of anti-integrin β1 antibody, but not anti-plexin C1 antibody. This suggests that integrin β1, rather than plexin C1, is responsible for the inflammatory response of HCAECs under KD conditions (Fig. 4b).

### Sema7A induces endothelial hyperpermeability through both plexin C1 and integrin β1

**Fig. 5:**
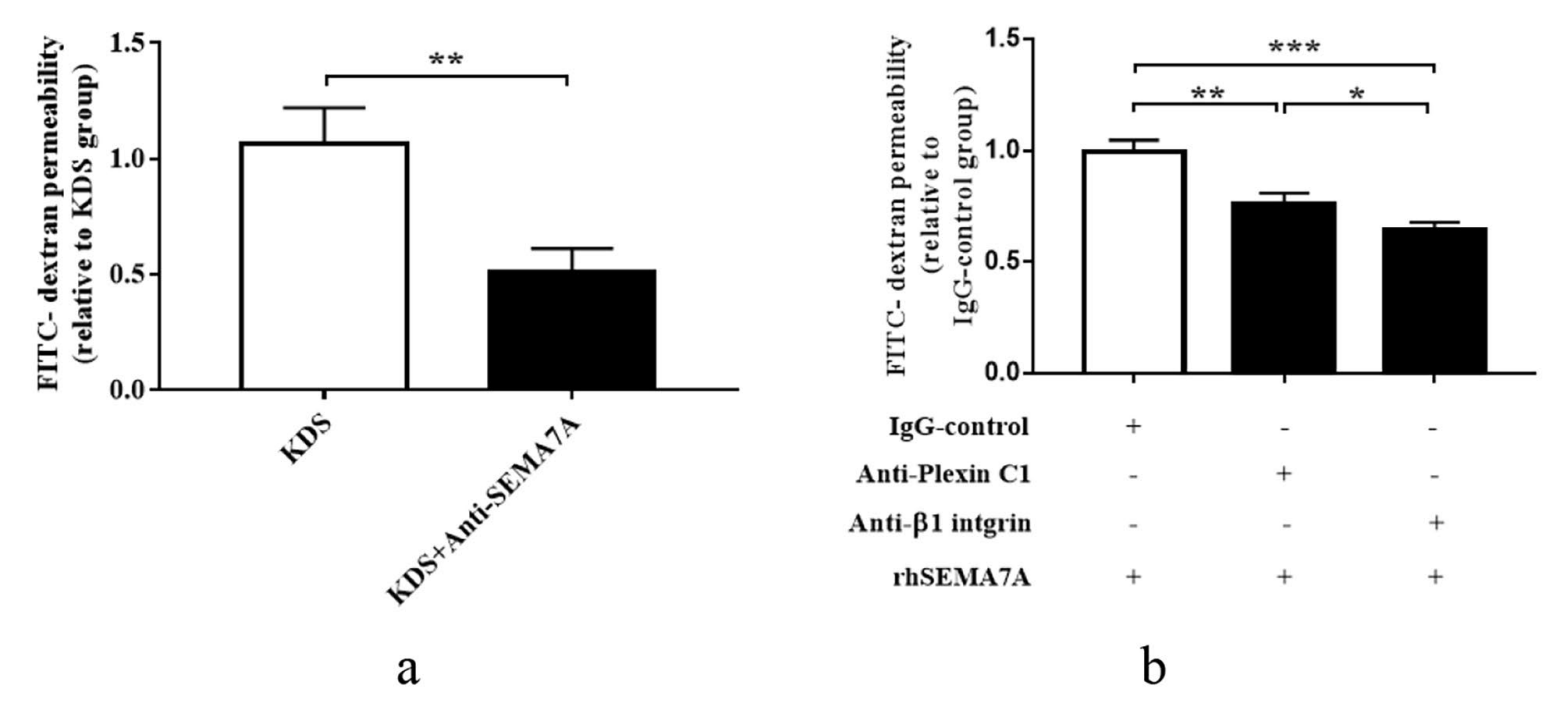
Sema7A induces HCAEC hyperpermeability via both plexin C1 and integrin β1. **a** Induction of HCAEC monolayer permeability by medium containing 20% untreated KD sera or 20% sSema7A-neutralized KD sera. **b** HCAEC permeability assay by rhSema7A stimulation upon blockade of plexin C1 and integrin β1, respectively. Data are expressed as M ± SEM from three experiments. *P < 0.05; **P < 0.01; ***P < 0.001. Sema7A: Semaphorin 7A; FITC: fluorescein isothiocyanate; KDS: sera from KD patients (medium containing 20% KD sera (pooled from 10 KD patients) ); KD: Kawasaki disease

Considering that KD sera can increase endothelial permeability compared to HC sera (as shown in Fig. 2b) and sSema7A is elevated in KD sera (as shown in Fig. 1a), we aimed to further illustrate the promoting role of Sema7A in endothelial permeability under KD conditions. To this end, we treated KD sera with anti-Sema7A antibody to neutralize sSema7A and then used the sSema7A-neutralized KD sera to induce endothelial permeability. We found that compared to untreated KD sera, sSema7A-neutralized KD sera had a weaker ability to promote HCAEC monolayer permeability (Fig. 5a). Next, to identify the receptor responsible for the hyperpermeability of the HCAEC monolayer induced by rhSema7A, we pretreated the HCAEC monolayer with anti-plexin C1 antibody and anti-integrin β1 antibody, respectively, followed by rhSema7A stimulation, and then measured FITC fluorescence intensity in the lower chambers. As illustrated in Fig. 5b, blocking both plexin C1 and integrin β1 significantly suppressed the rhSema7A-induced hyperpermeability of the HCAEC monolayer. It is noteworthy that blocking integrin β1 had a significantly stronger inhibitory effect on rhSema7A-mediated endothelial permeability than blocking plexin C1.

## Discussion

In this study, we demonstrate that ADAM17-mediated Sema7A promotes hyperpermeability and inflammation of HCAECs through both plexin C1 and integrin β1-dependent mechanisms in the context of KD. Our findings suggest that the Sema7A/plexin C1/integrin β1 axis is involved in the progression of KD, and Sema7A may serve as a novel prognostic and therapeutic candidate for KD vasculitis.

KD vasculitis is characterized by increased permeability and altered homeostasis of endothelial cells [19]. CAL is the most common complication of KD. However, the high incidence of CAL damage caused by KD is not fully understood, but researches suggest it may involve in immune response dysregulation, vascular endothelial injury, genetic susceptibility, inadequate or delayed treatment, and increased coronary blood flow pressure. In our study cohort, the high incidence of CAL is likely influenced by the unique characteristics of our patient population. As a national regional pediatric medical center in Northwest China, our hospital predominantly treats severe cases of KD. This preponderance of severe KD cases may contribute to the elevated CAL incidence, as patients with more severe manifestations are at a higher risk for cardiovascular complications. Furthermore, the geographical and socioeconomic context of our region plays a significant role in the healthcare challenges faced by our patients. The remote locations and potential limitations in healthcare access for some children in the region may result in delayed diagnosis and treatment, which are known factors that can increase the risk of CAL development. Endothelial activation plays a significant role in the pathophysiology of KD, but the underlying mechanism remains incompletely understood. In this study, we demonstrate that sSema7A levels are elevated in KD patients, resulting from ADAM17-mediated shedding of monocyte mSema7A, and this elevation is associated with disease severity. Furthermore, we observe that sera from KD patients upregulate the expression of plexin C1 and integrin β1 in HCAECs, which contributes to Sema7A-induced endothelial hyperpermeability and inflammation. These findings highlight the significant pathological role of Sema7A in KD and suggest its potential as a prognostic marker and therapeutic target in the disease.

Increasing evidence has demonstrated the crucial role of Sema7A in immune inflammatory responses [20]. Changes in sSema7A levels in the bloodstream have been observed in various autoimmune diseases [21] and cardiovascular disorders [7]. For instance, elevated levels of sSema7A have been reported in patients with rheumatoid arthritis (RA) [14]]. Conversely, decreased serum levels of sSema7A have been observed in systemic lupus erythematosus (SLE) and Sjögren’s syndrome (SS) [21]. Studies focusing on cardiovascular diseases have also shown significantly higher blood sSema7A levels in patients with acute atherothrombotic stroke (AAS) [22] and myocardial ischemia-reperfusion injury (MIRI) [23]. These findings suggest that alterations in sSema7A levels in the blood may be specific to certain diseases. In our study, we observed a significant increase in sSema7A levels in the serum of KD patients, particularly in those with CAL. This finding is consistent with the results obtained in RA [14] and cardiovascular diseases [23], indicating a potential common phenomenon of increased sSema7A in circulation in diseases associated with immune inflammation and cardiovascular damage. Interestingly, a recent study focusing on children with abdominal compartment syndrome (ACS), a life-threatening inflammatory condition, also demonstrated elevated serum Sema7A levels, which decreased after effective treatment [24]. This finding aligns with our observations in KD and suggests that Sema7A may have a broad involvement in inflammatory diseases in children. Furthermore, we observed a positive association between sSema7A levels and the severity of KD. Similarly, blood sSema7A concentration has been found to be associated with the risk of AAS [22] and the disease activity of RA [14]. Notably, our previous study demonstrated a positive correlation between Sema4D and CRP levels and Z-score in KD [10], suggesting that molecules from the Sema family may serve as progression markers in the development of KD vasculitis.

Regarding the cellular source of increased sSema7A in KD, we performed flow cytometry analysis to evaluate the surface expression of mSema7A on various leukocytes. Interestingly, we observed a significant decrease in mSema7A expression on monocytes, while no significant changes were observed on granulocytes and T cells. Importantly, we found a positive correlation between serum sSema7A concentration and circulating monocyte counts (Fig. 2c). A study by Xie et al. [14], conducted on RA, showed that both T cells and monocytes were identified as contributors to elevated sSema7A levels. However, in our study, we observed an increase in mSema7A expression on T cells (Fig. 2b), indicating a disease-specific characteristic of mSema7A shedding by different cell types. Several metalloproteases have been implicated in mSema7A shedding. In our study, we investigated the effect of two important KD-associated metalloproteases, MMP9 and ADAM17, on mSema7A cleavage. We found that ADAM17, but not MMP9, promoted the shedding of mSema7A from monocytes. Furthermore, the use of an ADAM17 inhibitor significantly suppressed the spontaneous shedding of mSema7A from monocytes. Additionally, we observed a positive relationship between ADAM17 expression and sSema7A levels in the blood of KD patients. It is worth noting that ADAM17 gene polymorphism has been reported in KD [25], and we have previously observed that ADAM17 can also cleave Sema4D on neutrophils [10], suggesting a crucial role of ADAM17 in KD, which warrants further investigation in future studies. It is worth noting that while our preliminary data and existing literature suggested that monocytes could be potential sources of Sema7A, our findings do not conclusively identify them as the primary sources. The immune response in KD is a complex and multifaceted process that involves the activation of a diverse array of immune cells. It is now increasingly recognized that beyond monocytes, other cell types such as granulocytes, T lymphocytes, B cells, dendritic cells, and even endothelial cells may play significant roles in the pathogenesis of KD. Each of these cell types has the potential to contribute to the serum levels of Sema7A through various mechanisms, including direct production, modulation of the inflammatory milieu, and interactions with other immune cells. The interplay between these cells and their microenvironment is crucial in shaping the immune response and the resulting levels of Sema7A. Therefore, we should take into account the potential heterogeneity of Sema7A sources, and we cannot rule out the contribution of other immune cell types to the elevated levels of Sema7A observed in the sera of KD patients. Taken together, our findings suggest that monocytes, at least in part, are responsible for the increased sSema7A levels in KD, and this process is mediated by ADAM17-mediated cleavage.

Sema7A has been shown to have proinflammatory effects on endothelium [26, 27]. However, the effect of Sema7A on HCAECs in KD situation is still unknown. In this study, we firstly stimulated HCAECs cultured in standard PRMI medium with 10% FBS using rhSema7A. Unexpectedly, we did not observe any significant differences in endothelial phenotypes between the rhSema7A stimulation group and the control group (Fig. 3a). A study from Hu et al. [7] has shown that Sema7A expression in mouse carotid artery is minimal, but it is markedly upregulated under disturbed flow conditions. Additionally, another study demonstrated an increased expression of Sema7A receptor in periapical lesions compared to normal conditions [28]. These findings suggest that the expression of Sema7A and its receptors can be altered under certain disease settings. Therefore, we speculated that in KD conditions, the expression of Sema7A receptors may also be changed. Considering that sera from KD patients have been used to stimulate HCAECs in numerous studies to mimic KD-conditioned endothelial phenotypes [29], and KD sera have been shown to alter the expression of endothelial RNA [30] and proteins [31], we cultured HCAECs with medium containing 20% KD sera. Intriguingly, we observed a significant upregulation of both plexin C1 and integrin β1 in HCAECs. In fact, inflammatory conditions have been shown to lead to differential expression of semaphorins and their receptors, as demonstrated by a study by Vreeken et al. [32]. Collectively, our findings suggest that Sema7A may have different effects on endothelial cells, and the expression of Sema7A receptors can be altered under disease conditions such as KD. These observations underscore the importance of studying the specific effects of Sema7A in different disease contexts.

Based on the KD cell model with upregulated plexin C1 and integrin β1 and the important role of inflammatory activation of vascular endothelial cells in KD vasculitis, we explored the action of Sema7A on inflammation in HCAECs. Our results showed that sSema7A promotes the overexpression and release of proinflammatory cytokines in HCAECs, a process dependent on integrin β1 but not plexin C1. By binding to Sema7A, integrin β1 exerts various biological functions in different cells, such as promoting endothelial to mesenchymal transition [33], mediating angiogenesis, regulating the expression of adhesion molecules [34], and stimulating the production of TNF-α in epithelial cells [35]. In this study, we demonstrated that the Sema7A/integrin β1 interaction promotes the production of TNF-α, IL-1β, IL-6, and IL-18 in HCAECs under KD context, suggesting a potent proinflammatory role of the Sema7A/integrin β1 axis in a wide range of cells. Interestingly, IL-1β and IL-18 are two important molecules in the process of nucleotide-binding oligomerization domain-like receptor family pyrin domain-containing 3 (NLRP3) inflammasome-mediated pyroptosis [36]. Whether the Sema7A/integrin β1 axis is involved in HCAEC pyroptosis deserves further investigation in the future. On the other hand, vascular hyperpermeability, which initiates with endothelial barrier injury, plays crucial roles in KD progression [37]. Sema7A has been reported to increase microvascular permeability. Zhang et al.’s [38] study on seawater aspiration-induced acute lung injury showed that Sema7A induces the expression of vascular endothelial growth factor (VEGF), a well-known endothelial permeability-related protein, and promotes hyperpermeability of pulmonary microvascular endothelial cells by interacting with plexin C1, while a study from Hu et al. [34] focused on atherosclerosis showed that Sema7A promotes VEGF-mediated endothelial permeability via interaction with integrin β1, suggesting a disease-context dependent feature of Sema7A. In this study, we found that neutralizing Sema7A with anti-Sema7A antibody significantly reduced KD sera-induced endothelial hyperpermeability (Fig. 5a). Furthermore, we showed that both plexin C1 and integrin β1 contribute to Sema7A-mediated HCAEC monolayer hyperpermeability in KD conditions (Fig. 5b), which expands our understanding of Sema7A-mediated vascular barrier injury and further indicates that Sema7A exerts its functions dependent on disease context.

There are several limitations that should be acknowledged in this study. Firstly, this study lacks in vivo experiments to explore the roles of Sema7A in KD vasculitis. Secondly, the sample size in this study is relatively small, and the inherent heterogeneity of serum composition may weaken the robustness of our conclusions. Therefore, further in vivo studies using KD mouse models and larger clinical studies are necessary. Thirdly, this present study was not able to fully characterize all cellular sources of Sema7A. While our data suggest a decrease in mSema7A expression on monocytes, this finding does not conclusively establish their role as a primary source of sSema7A. The complexity of the immune response in KD, characterized by the activation of multiple cell types, may involve a more intricate interplay of sSema7A production. Our study’s design, which focused on the analysis of peripheral blood monocytes, may not fully capture the dynamic changes in Sema7A expression across the entire immune system during KD. Future research should include an assessment of other potential Sema7A-producing cell types to provide a comprehensive understanding of Sema7A generation in KD and utilize advanced techniques such as single-cell RNA sequencing to dissect the cellular origins of sSema7A in greater detail. Additionally, functional assays to directly measure sSema7A production by different immune cells under KD conditions would provide further insights into the mechanisms underlying the observed changes in sSema7A levels.

## Conclusions

In summary, this study provides novel insights into the pathological roles of Sema7A and its receptors, plexin C1 and integrin β1, in HCAEC activation under KD conditions, specifically in relation to endothelial permeability and cytokine production. These findings suggest that Sema7A has the potential to serve as a novel prognostic marker and therapeutic target for KD.

## Data Availability

The data supporting the findings of this study are available from the corresponding author upon request.

## Abbreviations

KD: Kawasaki disease
Sema7A: Semaphorin 7 A
ADAM17: A Disintegrin and Metalloprotease 17
HCAECs: Human coronary artery endothelial cells
CAL: coronary artery lesions
IVIG: intravenous immunoglobulin
MMP9: matrix metallopeptidase 9
PBMCs: Peripheral blood mononuclear cells
RPMI: Roswell Park Memorial Institute
FBS: fetal bovine serum
qRT-PCR: quantitative real-time polymerase chain reaction
PBS: phosphate buffer solution
CRP: C reaction protein
Alb: albumin
